# Supplementary material for: Benefits of Valsartan and Amlodipine in Lipolysis through PU.1 Inhibition in Fructose-Induced Adiposity
Source: Nutrients. 2022 Sep 12;14(18):3759. doi: 10.3390/nu14183759 (PMC9502698; doi:10.3390/nu14183759)

Supplementary Materials

Figure S1. Experimental protocol for cell cultures.

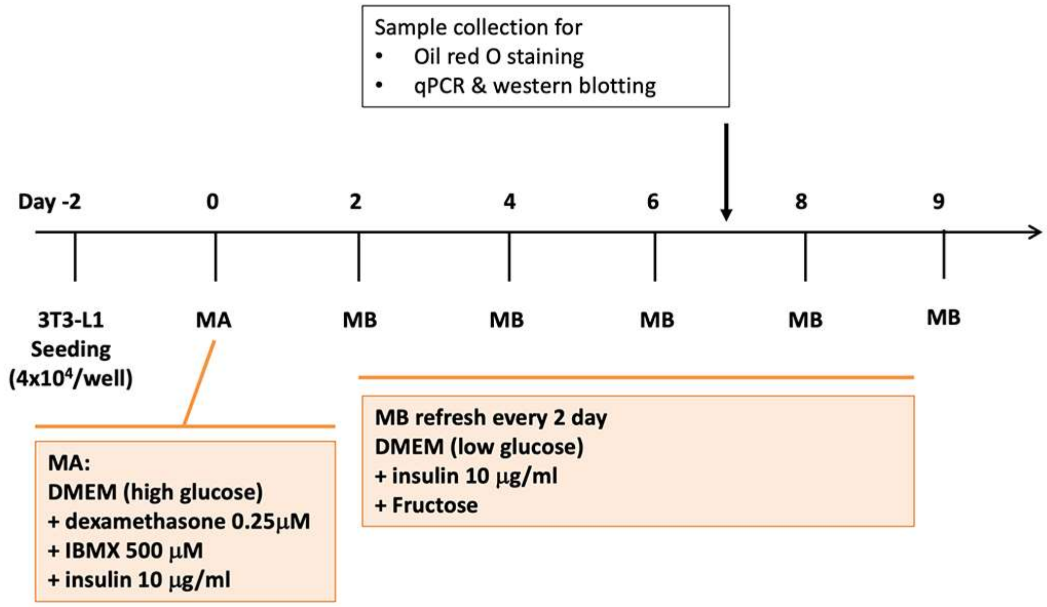

Figure S2. Effects PU.1 inhibitor, valsartan, and amlodipine on the protein levels of ATGL, SCD1 in adipocytes in vitro.

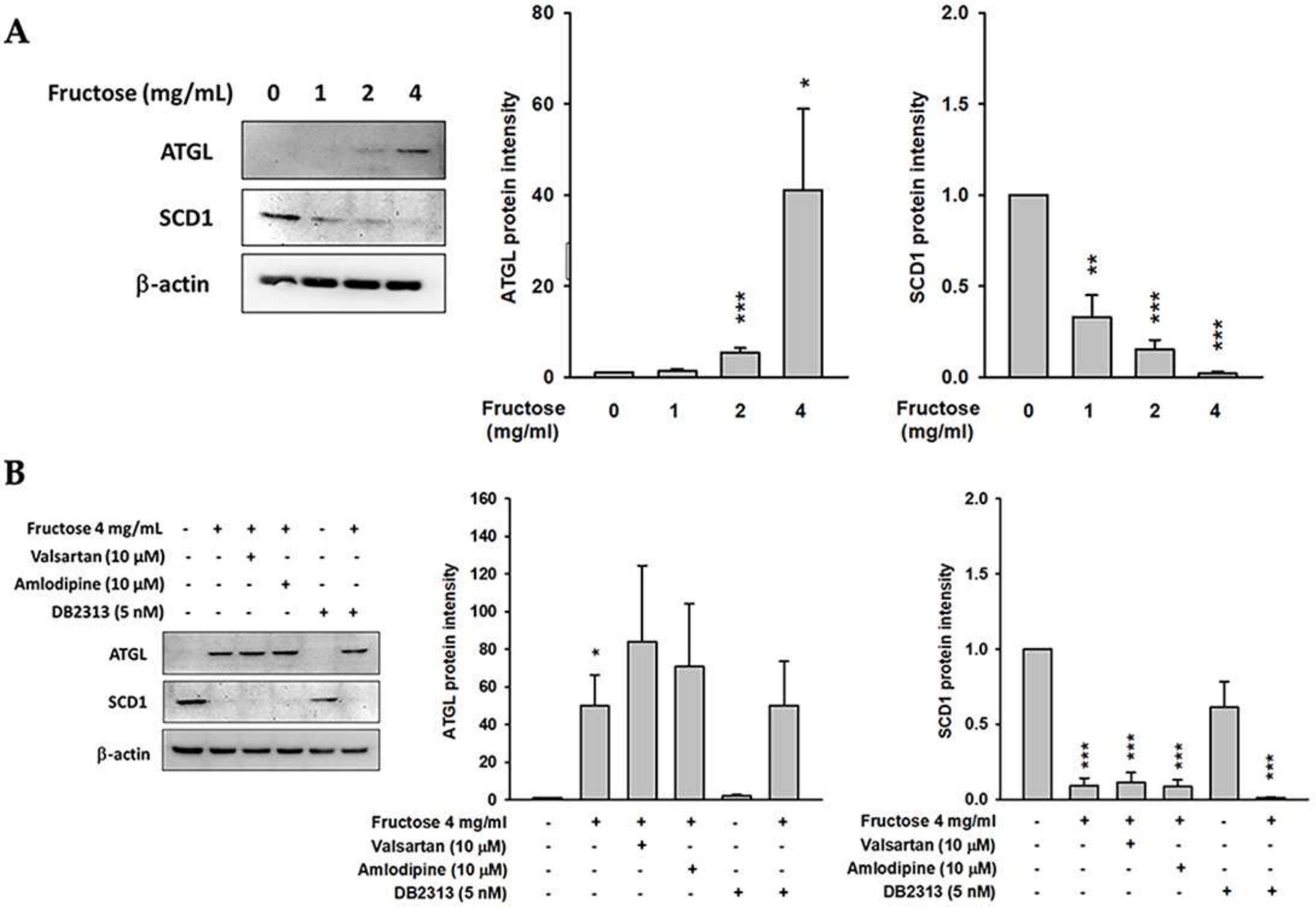

Supplement: Supplementary file 1 [file nutrients-14-03759-s001.zip › nutrients-1871632-supplementary.pdf]
